# Controlled Release of Epidermal Growth Factor from Furfuryl-Gelatin Hydrogel Using in Situ Visible Light-Induced Crosslinking and Its Effects on Fibroblasts Proliferation and Migration

**DOI:** 10.3390/gels8040214

**Published:** 2022-04-01

**Authors:** Min Sun Kong, Won-Gun Koh, Hyun Jong Lee

**Affiliations:** 1 Department of Chemical and Biological Engineering, Gachon University, 1342 Seongnam-daero, Seongnam-si 13120, Korea; minsunlab@gmail.com; 2 Department of Chemical and Biomolecular Engineering, Yonsei University, 50 Yonsei-ro, Seodaemun-gu, Seoul 03722, Korea

**Keywords:** gelatin hydrogel, furfuryl group, photocrosslinking, riboflavin phosphate, epidermal growth factor

## Abstract

Hydrogels are widely used in tissue engineering as materials that regulate cell proliferation, migration, and differentiation. They also act as promising biomaterials that can provide a variety of stimuli by influencing the surrounding microenvironment, which can be achieved by modulating their mechanical properties, thereby aiding soluble factor delivery. Here, we developed a gelatin-based injectable hydrogel that has controllable mechanical properties and demonstrates sustained drug release without the need for invasive surgery. Gelatin was modified with furfuryl groups, and riboflavin phosphate was used as a photoinitiator to crosslink the hydrogel using visible light. A hydrogel–with a storage modulus in the range of 0.2–15 kPa was formed by maintaining the concentration of furfuryl-gelatin within 10–30% *w/v*. Consequently, their mechanical properties can be tailored for their applications. The furfuryl-gelatin hydrogel was loaded with maleimide-modified epidermal growth factor (EGF) as a model drug to achieve a controlled-release system. The sustained release of maleimide-EGF due to gelatin hydrogel matrix degradation was observed. Cell proliferation and scratch assays were performed to verify its effect on fibroblasts. When EGF was physically entrapped in the hydrogel matrix, the released EGF considerably affected cell proliferation and scratch closure of fibroblasts at the beginning of the culture. By contrast, maleimide-EGF was released sustainably and steadily and affected cell proliferation and scratch closure after the initial stage. We demonstrated that the release of soluble factors could be controlled by modulating the mechanical properties. Thus, the injectable hydrogel formed by in situ visible light-induced crosslinking could be a promising biomaterial for tissue engineering and biomedical therapeutics.

## 1. Introduction

Hydrogels are among the most widely used materials in biomedicine. This versatile and biocompatible material comprises a three-dimensional water matrix that mimics the extracellular matrix (ECM) [1,2]. Thus, it is considered a promising material for tissue engineering. The physical and chemical properties of a hydrogel vary depending on the unit material and polymerization method. Many studies have investigated the interaction between cells and cell proliferation, migration, and differentiation using hydrogels [3,4,5]. Hydrogels can be prepared using synthetic or natural polymers [6,7]. Synthetic polymers, such as polyethylene glycol and polyurethane, have the advantage of controlling the microstructure and decomposition rate as their chemical modification and physical property adjustments are relatively simple, but they lack biological moieties [7,8]. By contrast, representative natural polymers, such as collagen and hyaluronic acid (HA), have good biocompatibility and biodegradability as they are composed of the original tissue material. Nonetheless, drawbacks, such as the lack of mechanical properties and difficulty in tuning the structure and degradability, limit their biomedical applications [7]. Therefore, three-dimensional hydrogels of natural polymers with the enhanced mechanical strength and controllable degradation will be more useful in the tissue engineering field.

Gelatin, a natural biopolymer, is partially hydrolyzed collagen obtained by physical or chemical decomposition [9]. It has the same chemical composition as collagen, which is a major component of the connective tissues in the body. Therefore, gelatin is an ideal biomaterial, with excellent biodegradability and biocompatibility [10]. It is more readily soluble in water than collagen and has unique characteristics, such as sol-gel transition regarding temperature. The gelatin solution forms a physically crosslinked hydrogel near 25 °C, which dissolves above 60 °C [11]. However, physically crosslinked gelatin hydrogels degrade quickly near body temperature and have poor mechanical properties, thus challenging their handling and maintenance. Therefore, the covalently crosslinked gelatin hydrogel formed by chemical reactions are more practical for tissue engineering applications. Various chemical crosslinking methods involve crosslinking using methacrylate, dynamic crosslinking, or dual crosslinking [1,11,12,13,14]. Furthermore, the applicability of injectable hydrogels in tissue engineering is broader than that of typical precured hydrogels. Therefore, an in situ crosslinked hydrogel formed through light irradiation after injection of the precursor solution is warranted. For example, GelMA, a gelatin modified with a methacrylate group, is a widely used gelatin that uses UV light as a primary source for photoinitiation [1]. However, UV light can cause cell damage in living organisms, causing problems such as skin cancer, genetic mutations, and a weakened immune system [15]. Visible light causes fewer adverse effects than UV light; therefore, it is more suitable for direct use in living organisms.

The furfuryl groups, having a chemical structure composed of a furan ring, are crosslinked by using photoinitiation [16,17,18,19]. A combination of a photoinitiator and UV light induces crosslinking between the furfuryl groups; however, visible light and appropriate photoinitiators can also be used to achieve crosslinking. The photoinitiators available for the visible light-induced crosslinking reaction include riboflavin, eosin Y, rose bengal, and methylene blue, and among them, riboflavin has the lowest cytotoxicity [20]. Riboflavin initiates the reaction by inducing the formation of singlet oxygen using blue light in the visible spectrum. Its utilization is not only limited to furfuryl group-based crosslinking but also extended to the thiol-ene reaction and crosslinking of collagen [21,22]. Riboflavin absorbs visible light energy to form a singlet-excited state and then immediately converts into a highly reactive triplet-excited state. The reactive oxygen is delivered to the furfuryl group to induce bonding between the furfuryl groups [18]. Some researchers used visible light irradiation to crosslink GelMA, but triethanolamine is required as a coinitiator. Furfuryl-gelatin is better suited to the simple crosslinking method under visible light [23,24].

Chemical crosslinking increases the mechanical strength of the hydrogel, thereby not only facilitating handling but also affecting the behavior of surrounding tissues and mechanotransduction [4]. In general, a hydrogel with mechanical properties similar to those of the target tissue induces normal tissue cell function [4,25]. Therefore, they are adjusted and fabricated to provide appropriate stiffness to the target tissue. However, it is difficult to completely control the cell microenvironment by changing the mechanical properties of the hydrogel. Hydrogels must be loaded with soluble factors, such as growth factors, to form an ideal cellular microenvironment. Generally, a long-term release with an appropriate amount of soluble factor is usually required, but soluble factors encapsulated in a biopolymer hydrogel matrix with a large molecular weight demonstrate an initial burst release profile. Therefore, designing the delivery system to achieve sustained release at an optimal rate is also essential [18,26,27].

Epidermal growth factor (EGF), a growth factor that affects cell growth and differentiation, is a representative soluble factor mounted on hydrogels. Owing to the low molecular weight of EGF (approximately 6000 mol/g), it is released rapidly by diffusion when physically entrapped in the hydrogel matrix [28,29]. To achieve a sustained release profile, EGF is chemically bound to a matrix by coupling with a functional group [30]. The maleimide functional group selectively reacts with the furfuryl group via the Diels–Alder reaction under mild conditions [11]. Therefore, the sustained release of soluble factors can be induced by introducing functional groups, such as maleimide and furfuryl groups, that form selective coupling for binding soluble factors and the hydrogel matrix.

In this study, a gelatin hydrogel was prepared by using the furfuryl group-modified gelatin and crosslinking it by irradiation with riboflavin phosphate and blue light. As a result, we achieved controlling physical property and sustained release of growth factors necessary for tissue-engineering applications. The mechanical property and degradation rate were controlled with a crosslinking density of furfuryl-gelatin hydrogel. The possibility of preparing an in situ, injectable gelatin hydrogel with controllable mechanical properties and crosslinked by photoinitiation was assessed for biomedical applications. In addition, the maleimide group-modified EGF was chemically bound to the gelatin matrix through a Diels–Alder reaction, and the resulting EGF-release behavior was monitored. By evaluating the effect of the drug release behavior on the proliferation and migration of fibroblasts, we verified the usability of an injectable, in situ gelatin hydrogel in tissue engineering.

## 2. Results and Discussion

### 2.1. Synthesis and Characterization of Furfuryl-Gelatin

**Figure 1:**
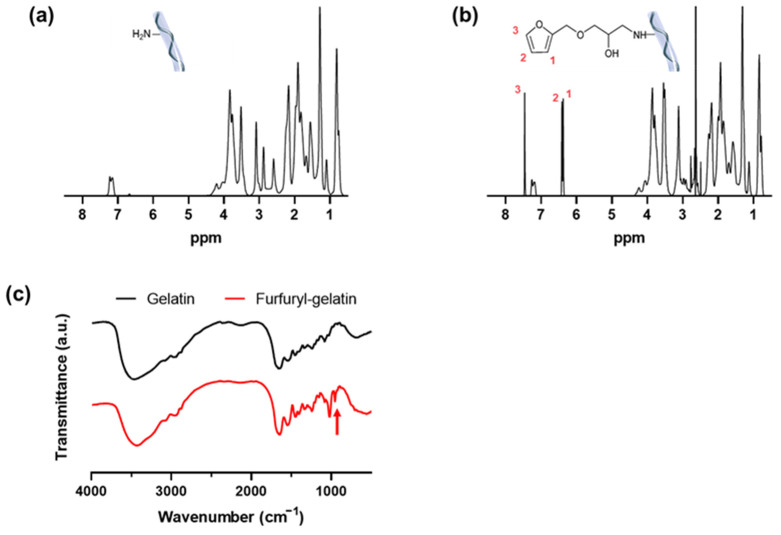
^1^H-NMR spectra of (**a**) gelatin and (**b**) furfuryl-gelatin. (**c**) FT-IR spectra of gelatin and furfuryl-gelatin.

Gelatin is a valuable material owing to its maintained collagen components, strong water solubility at physiological pH, sol–gel transition at 25–30 °C, and existence in the form of a gel at lower temperatures [9,10,11]. However, chemical crosslinking is essential to increase the usability of gelatin as it exists in a liquid phase at body temperature and has limited mechanical strength. In this study, a hydrogel was fabricated by crosslinking gelatin with a furfuryl group. The furfuryl group is a visible light-reactive functional group and photo-oxidation crosslinking is achieved by using an appropriate photoinitiator and visible light irradiation [16,17]. A furfuryl functional group was introduced into gelatin by coupling furfuryl glycidyl ether with amine groups present in lysine, hydroxylysine, and the N-terminus of gelatin, as described previously [11,14]. The ^1^H-NMR peaks at 6.37, 6.42, and 7.46, indicating the presence of the furan ring, thereby establishing the presence of the furfuryl group in gelatin (Figure 1a and b). The peak area of the furan signal was measured, and the furan group was about 2.87 mol% in furfuryl-gelatin. The content of lysine residues in gelatin is about 3.50 mol% [31]. Therefore, the estimated degree of substitution by ^1^H-NMR was approximately 82.0%.

Furthermore, furfuryl-gelatin was compared with gelatin by using FT-IR spectroscopy analysis (Figure 1c). The freeze-dried gelatin and furfuryl-gelatin were sampled and measured using a KBr pellet. At 1630 and 1540 cm^−1^, two amide peaks common to gelatin and furfuryl-gelatin were observed. Amide I (1630 cm^−1^) peak represents the C=O stretching vibration and amide II (1540 cm^−1^) peak represents the N–H bending and C–N stretching vibrations. In furfuryl-gelatin, a prominent peak was observed at 920 cm^−1^ and not seen in gelatin. It is a representative peak of the furan ring, which indicates =C–H deformation vibration [11]. These results further demonstrate that the furfuryl group was integrated into gelatin via chemical bonding.

### 2.2. Fabrication and Characterization of Visible Light-Induced, Crosslinked Furfuryl-Gelatin Hydrogel

**Figure 2:**
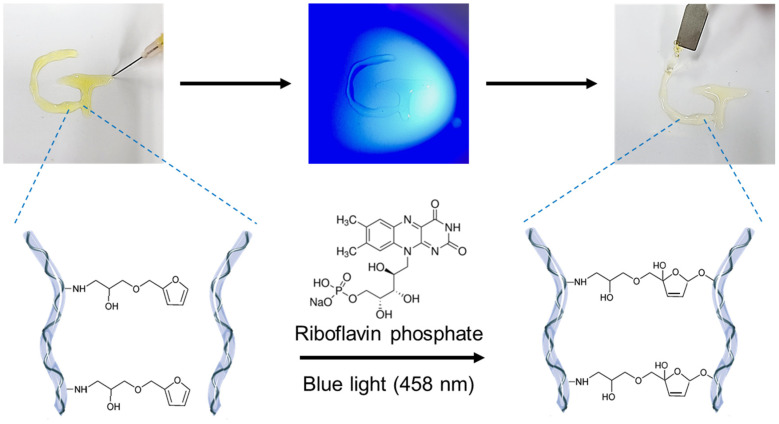
Photocrosslinking mechanism of furfuryl-gelatin hydrogel by blue light (458 nm) irradiation using riboflavin phosphate.

The furfuryl group has been used to fabricate hydrogels by crosslinking with biopolymers, such as gelatin, alginate, chitosan, and HA [14,16,17,18,19,20]. In the present study, a hydrogel was formed by adding riboflavin phosphate as a photoinitiator to the furfuryl-gelatin solution and exposing it to blue light (Figure 2). In the presence of singlet oxygen, furfuryl groups crosslink through the formation of furan endoperoxides [14,16,18]. The singlet oxygen can be formed by various photoinitiators using light sources of different wavelengths. Some photocured materials often use UV light, but the use of visible light can reduce cytotoxicity and harm to the human body [15,32]. The riboflavin phosphate used in this study is a water-soluble form of riboflavin. The US FDA approved its use in 2016 as a material for crosslinking collagen when used with UV light [21]. Riboflavin phosphate generates singlet oxygen under UV and blue light with a wavelength of 458 nm; the latter is safer for use in humans [22].

**Figure 3:**
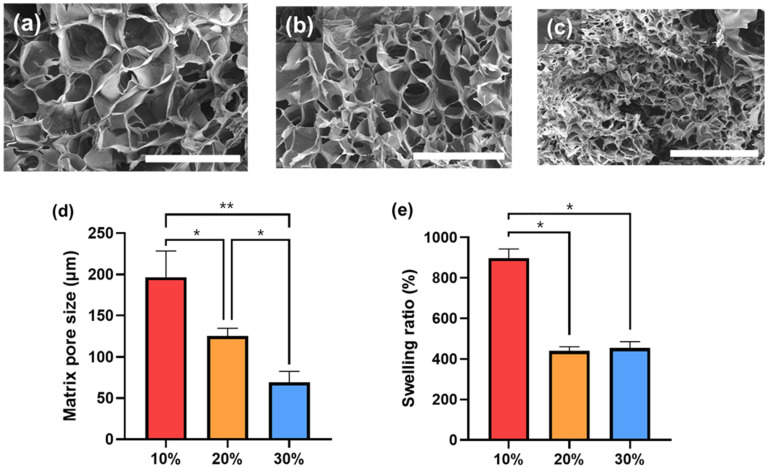
Scanning electron microscopy (SEM) images of (**a**) 10%, (**b**) 20%, and (**c**) 30% furfuryl-gelatin hydrogels. Scale bars are 500 μm. (**d**) Matrix pore sizes of furfuryl-gelatin hydrogel matrices (n = 10). (**e**) Equilibrium swelling ratio of furfuryl-gelatin hydrogels. The significant differences are represented by 0.01 < *p* < 0.05 (*) and *p* < 0.01 (**).

The matrix morphology of the freeze-dried photocrosslinked furfuryl-gelatin hydrogel was investigated using scanning electron microscopy (SEM) (Figure 3a–c). The hydrogels had porous and interconnected structures. Although the shape of the pores did not show significant differences, the change in the pore size with concentration was evident. The average pore sizes of the 10%, 20%, and 30% furfuryl-gelatin hydrogel matrices were 196 ± 26 μm, 125 ± 8 μm, and 69 ± 11 μm, respectively (Figure 3d). As the concentration increased, the amount of gelatin and furfuryl groups constituting the matrix increased. By contrast, the pore size decreased as the number of linking sites per unit area increased.

Physical properties, such as hydrogel degradation and drug release rate, were affected by the water content of the hydrogel. Thus, their weight was measured after soaking the fabricated furfuryl-gelatin hydrogel in phosphate-buffered saline (PBS) for 24 h. Then, the swelling ratio was calculated by measuring the weight of lyophilized hydrogel (Figure 3e). The swelling ratio of the 10% furfuryl-gelatin hydrogel was ~900%. By contrast, 20% and 30% furfuryl-gelatin showed similar swelling ratios of approximately 450%. After 24 h, the measurement of the swelling ratio was attempted, but handling was impossible due to hydrogel degradation. In general, smaller pore size of the hydrogel matrix results in higher crosslinking density, which, in turn, lowers the swelling ratio [2]. The 30% furfuryl-gelatin hydrogel precursor solution has a higher viscosity than the low concentration solution; therefore, the interaction between furfuryl-gelatin molecules might not be active when the reaction occurred. Thus, the modified furfuryl groups of gelatins would not sufficiently crosslink, resulting in a low crosslinking density with a low swelling ratio, similar to that of the 20% furfuryl-gelatin hydrogel.

### 2.3. Mechanical Properties of the Crosslinked Furfuryl-Gelatin Hydrogel

**Figure 4:**
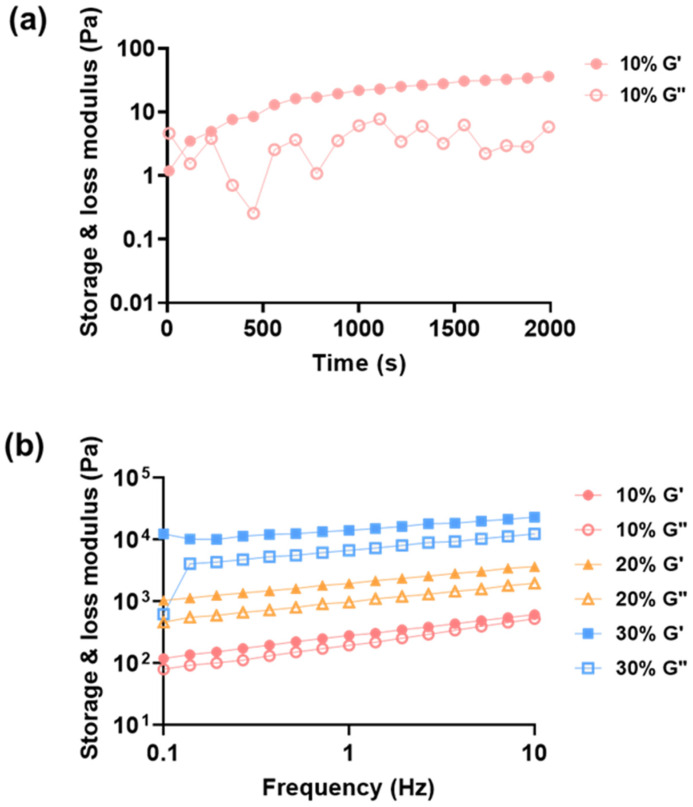
Variation of dynamic moduli (G’: storage moduli, G”: loss moduli) of furfuryl-gelatin hydrogels with (**a**) time for 10% furfuryl-gelatin and (**b**) frequency for 10%, 20%, and 30% furfuryl-gelatin.

The dynamic moduli of the photocrosslinking process of furfuryl-gelatin were measured using a rheometer. The storage modulus (G’) and loss modulus (G’’) were measured by irradiating a 10% *w/v* furfuryl-gelatin solution with blue light at 37 °C (Figure 4a). Before exposure to blue light, the solution was in a liquid state with loss modulus higher than the storage modulus. The gel point at which the storage and loss modulus intersect was observed around 120 s after irradiation with visible light. However, gelling point with the storage modulus similar to the loss modulus was observed on irradiation with the blue light. The storage modulus continued to increase as the crosslinking progressed. This rapid increase is observed until 600 s beyond which the increment rate became moderate, signifying the completion of crosslinking. Based on these findings, the experiment was performed by setting the blue light irradiation time to 600 s.

Among the variety of physical properties, the mechanical property of hydrogels is the most important for their utility in tissue-engineering applications. Here, the storage moduli of the furfuryl-gelatin hydrogel (10–30% *w/v*) were measured to determine the range of adjustable mechanical properties of the hydrogels based on the furfuryl-gelatin concentration. After dissolving furfuryl-gelatin in PBS, solutions were prepared to contain 0.01% *w/v* riboflavin phosphate. Subsequently, blue light was irradiated for 600 s and incubated at 37 °C for 3 h to complete crosslinking (Figure 4b). The hydrogels were mounted on the stage of the rheometer, and the dynamic moduli were measured. There was sample slippage when the high frequency was applied, thus the data under 10 Hz was plotted. The storage moduli of the 10%, 20%, and 30% hydrogels were 283 ± 44 Pa, 1995 ± 259 Pa, and 14,369 ± 1369 Pa, respectively. It is clear from the observations that the higher concentrations result in stiffer hydrogels with higher elastic moduli.

The storage modulus increased approximately seven times as the concentration was increased by 10%. The hydrogel with a concentration of less than 10% was difficult to handle; therefore, 10% was used as the lowest concentration for the experiment. The highest concentration used in this experiment was 30%, as this solution was highly viscous. Beyond this concentration of furfuryl-gelatin, hydrogel becomes uninjectable. The approximate range of the storage modulus of the hydrogel was approximately 0.2–150 kPa for the controlled concentration range. The storage modulus cannot be directly compared to the Young’s modulus; therefore, we estimated the elastic modulus using the following equation:

E = 2G (1 + ν)(1)

where E is the Young’s modulus, G is the storage modulus, and ν is Poisson’s ratio [33]. Poisson’s ratio is a constant that depends on the material and has been reported to be ≈0.45 for collagen fibers [34]. The Young’s modulus becomes approximately 0.6–45 kPa as the storage modulus varies from 0.2 to 150 kPa. When hydrogel is used as a scaffold, the physical properties of the hydrogel are more similar to tissue, and conditions more similar to the natural environment can be provided to cells. The elasticity of collagenous bone is approximately 100 kPa and osteogenesis was previously reported to be induced in a 40–kPa hydrogel substrate [25]. Therefore, our hydrogel can control a broad range of elasticity, from soft brain tissue to relatively rigid collagenous bone tissues.

**Figure 5:**
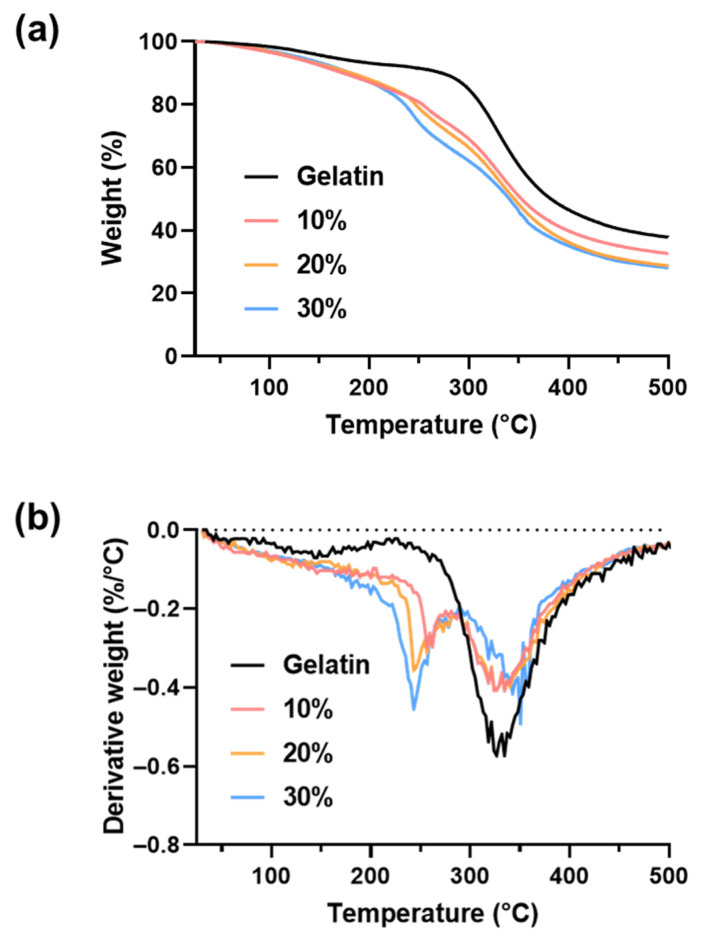
Thermal decomposition of gelatin and furfuryl-gelatin hydrogels. (**a**) Thermogravimetric (TG) curves. (**b**) Derivative thermogravimetric (DTG) curves.

The effect of the furfuryl group and the concentration of furfuryl-gelatin on the thermal stability of the hydrogel was investigated using thermogravimetric analysis (Figure 5). All samples were freeze-dried to conduct thermal analysis in the absence of water, and weight loss was measured as a function of temperature. As gelatin undergoes reversible sol–gel transition without chemical modification on increasing the temperature, its decomposition began at approximately 280 °C with rapid weight loss until 400 °C. By contrast, furfuryl-gelatin exhibited the first weight loss at 200–280 °C, followed by a second weight loss near 300 °C (Figure 5a). All the samples showed the same pattern regardless of furfuryl group introduction, with rapid weight loss up to 400 °C. Taking the differentiation of the weight loss graph, the decomposition peaks of the furfuryl-gelatin hydrogel were clearly divided into two, as shown in Figure 5b. Gelatin had a significant weight loss rate at approximately 320 °C, whereas furfuryl-gelatin had it twice at approximately 250 °C and 330 °C.

The early decomposition of the furfuryl-gelatin hydrogel at approximately 250 °C was due to the introduction of a furfuryl group. Poly(furfuryl alcohol) containing furfuryl groups exhibits a high degradation rate peak at approximately 210 °C [35]. When the furfuryl-gelatin concentrations were 10%, 20%, and 30%, the first maximum degradation rate peaks were at approximately 260, 245, and 240 °C, respectively. The higher the content of furfuryl-gelatin, the closer the degradation is to 210 °C and the higher the weight loss rate. The second peak corresponds to gelatin, which showed a significant decrease at approximately 330 °C, but the decrease in furfuryl-gelatin hydrogels was not as significant as in gelatin. In addition, as the furfuryl-gelatin content increased, the peak of the rate corresponding to gelatin degradation shifted toward a higher temperature. Therefore, the 30% furfuryl-gelatin hydrogel exhibited the maximum decomposition rate at approximately 350 °C. Thus, the high concentration furfuryl-gelatin hydrogel reduced gelatin matrix decomposition by increasing the crosslinking density, while also allowing the gelatin matrix to decompose at a higher temperature owing to its improved thermal stability.

**Figure 6:**
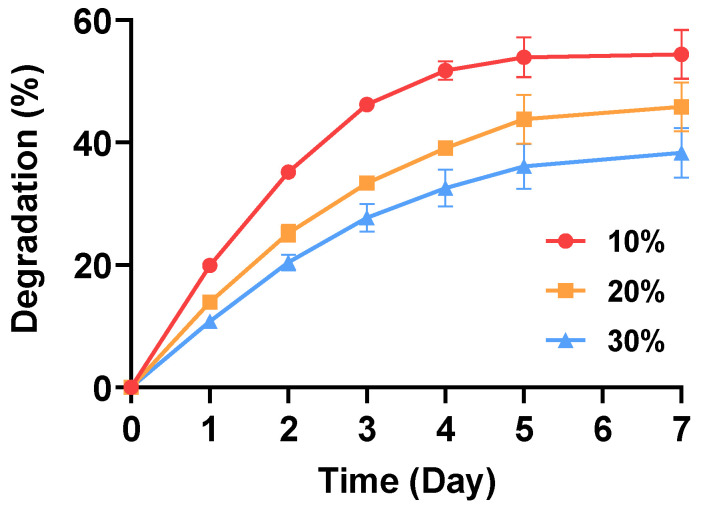
The degradation profiles of furfuryl-gelatin hydrogels.

The decomposition rate of the furfuryl-gelatin hydrogel was determined by measuring the amount of gelatin in the incubated solution through protein quantification (Figure 6). The lower the concentration of furfuryl-gelatin, the higher is the degradation rate. The total amounts of degradation after seven days were approximately 54%, 46%, and 38% for the 10%, 20%, and 30% furfuryl-gelatin hydrogels, respectively. Moreover, the matrix structure and degradation rate are related to each other; as the concentration of furfuryl-gelatin decreases, the porosity decreases, and the pore size increases. Therefore, the diffusion of an acidic degradation product is inhibited, resulting in stronger acid-catalyzed hydrolysis. Thus, the bigger the pores, the faster is the degradation rate [36,37].

### 2.4. Modification of EGF with the Maleimide Group and Its Incorporation in the Furfuryl-Gelatin Hydrogel

**Figure 7:**
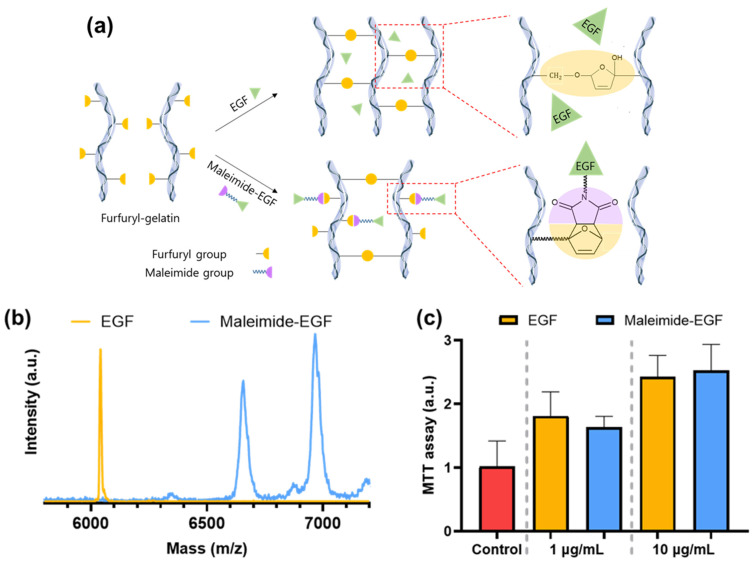
(**a**) Schematic representation of chemical coupling of maleimide-EGF with furfuryl-gelatin. (**b**) MALDI-TOF spectra of EGF and maleimide-EGF. (**c**) Cell proliferation due to EGF and maleimide-EGF.

EGF is a growth factor that aids the growth of fibroblasts and can provide optimal stimuli to cells through appropriate sustained release [28,29]. When a drug is applied to an injectable hydrogel, it is challenging to supplement it after injection; therefore, the incorporated drug must be controlled and released from the hydrogel for a long time. The molecular weight of EGF is approximately 6044 g/mol, and its size is smaller than the matrix pore size. Therefore, when EGF is mixed with the hydrogel precursor solution and physically confined in the hydrogel matrix, it releases within 1–2 days; therefore, its effect is very short lived [26,38]. Growth factors are released at a lower rate if EGF is chemically bound to the hydrogel matrix. Therefore, a maleimide group was introduced onto EGF to help it chemically bond to the furfuryl-gelatin hydrogel matrix. The maleimide and furfuryl group combination is a kind of Diels–Alder “click chemistry” and selective bonding [11]. Therefore, it was possible to selectively bind maleimide-EGF to furfuryl-gelatin (Figure 7a).

Specifically, maleimide-PEG_2_-NHS ester was incubated when a maleimide group was conjugated to EGF using the NHS ester coupling reaction. The introduction of the maleimide group to EGF was verified using MALDI-TOF (Figure 7b). The molecular weight increased by approximately 311 g/mol when one maleimide-PEG_2_ group was introduced into the maleimide-PEG_2_-NHS ester. The molecular weights of maleimide_1_-EGF, maleimide_2_-EGF, and maleimide_3_-EGF were 6355, 6666, and 6977 g/mol, respectively. Human EGF has three primary amines, one on the N-terminal and two on the side chain of lysines (Lys28 and Lys48). Among them, the N-terminal amine has higher nucleophilicity than lysine [39,40]. When maleimide-modified EGF was analyzed using MALDI-TOF, unmodified EGF and maleimide_1_-EGF were almost absent. Most EGF were modified into maleimide_2_-EGF and maleimide_3_-EGF by reacting two or three amine groups with maleimide-PEG_2_-NHS ester.

The degree of fibroblast proliferation induced by EGFs was compared at 1 and 10 µg/mL to investigate the bioactivity of maleimide-modified EGF. It was observed that the maleimide-EGF induced a similar level of fibroblast proliferation at both these concentrations (Figure 7c). This finding indicates that maleimide modification did not affect the bioactivity of EGF.

**Figure 8:**
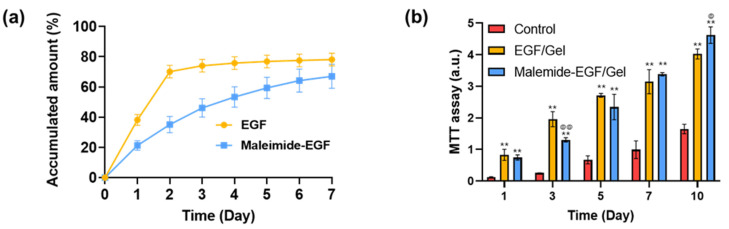
(**a**) Release profiles of EGF and maleimide-EGF from furfuryl-gelatin hydrogels. (**b**) Cell proliferation by EGF and maleimide-EGF incorporated in furfuryl-gelatin hydrogels. The symbols * and @ are used to represent significant differences when compared with the control and EGF/Gel groups, respectively. The significant differences are represented by 0.01 < *p* < 0.05 (@) and *p* < 0.01 (** and @@).

The difference in the EGF-release profiles was measured with respect to the modification of the maleimide group (Figure 8a). EGF/Gel and maleimide-EGF/Gel groups were prepared by adding 1 μL of EGF or maleimide-EGF solution (0.01 mg/mL) to 100 µL of the furfuryl-gelatin hydrogel precursor solution, respectively. The EGF or maleimide-EGF in the collected PBS solution was quantified using an EGF ELISA. Approximately 70% of EGF was released within two days with a burst release behavior when the hydrogel was formed by adding unmodified EGF to the furfuryl-gelatin hydrogel precursor solution. Conversely, maleimide-EGF release was constant with approximately 67% of the maleimide-EGF released until day seven. In addition, the release of maleimide-EGF was similar to the degradation profile of the furfuryl-gelatin hydrogel, which indicates that EGF is bound to the matrix; thus, it is released as the matrix undergoes degradation.

Next, we evaluated the effect of the sustained release of maleimide-EGF on cell proliferation (Figure 8b). The groups that included EGF or maleimide-EGF showed significantly increased proliferation compared to the control group. The maleimide-EGF/Gel group, where EGF was connected via chemical bonding with furfuryl-gelatin, was compared to the EGF/Gel group, wherein EGF was physically entrapped in the hydrogel matrix. There was no significant difference between the two groups on day one, but the fibroblasts in the EGF/Gel group showed a high proliferation ability on day three. As seen in Figure 8a, it is evident that the EGF/Gel group released a more significant amount of EGF than the maleimide-EGF/Gel group until day two. Thus, higher proliferation was induced by the addition of more EGF. After day five, the proliferation rate of cells in the maleimide-EGF/Gel group increased. On day 10, the cells in the maleimide-EGF/Gel group showed a statistically significant increase in proliferation. In the EGF/Gel group, the amount of unmodified EGF released from the gel after day two was small; therefore, the effect on the proliferation of fibroblasts was significantly reduced. By contrast, maleimide-EGF continuously stimulated cell proliferation by providing EGF to the hydrogel. When rapid cell proliferation is required, a large amount of growth factors can be easily provided through an initial burst release without chemical modification. However, chemical modification is required for sustained long-term stimulation, and we have demonstrated the usefulness of a combination of furfuryl and maleimide.

**Figure 9:**
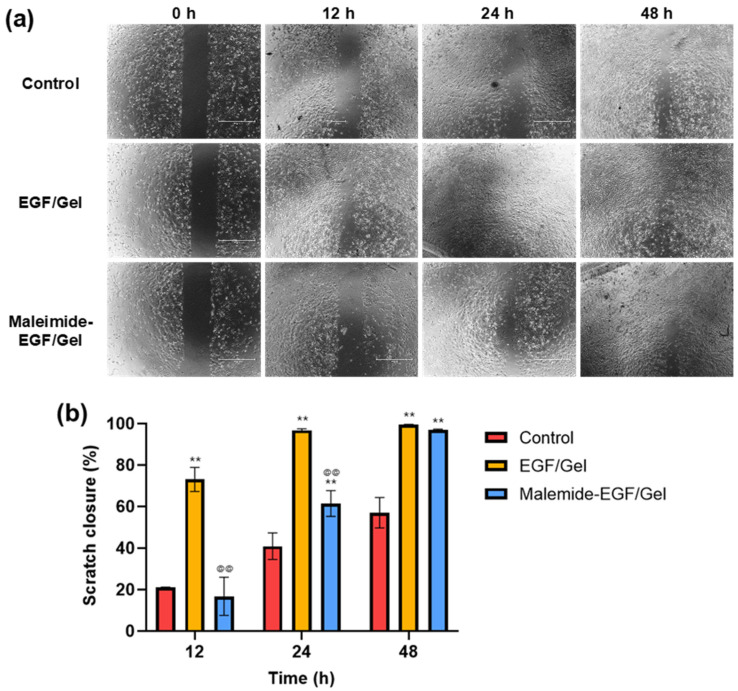
Scratch assay of fibroblasts for EGF and maleimide-EGF as a function of treatment conditions. (**a**) Representative images taken at 0, 12, 24, and 48 h after applying EGFs. (**b**) Quantification of scratch closure using the wound-healing measurement tool of ImageJ. The scratch closure was evaluated by dividing the open area by initial scratch area. The symbols * and @ are used to represent significant differences when compared with the control and EGF/Gel groups, respectively. The significant differences are represented by *p* < 0.01 (** and @@).

Additionally, the effect of the EGF-release profiles on fibroblasts was evaluated using a scratch assay (Figure 9). At 12 h after scratching, scratch closure in the EGF/Gel group was predominantly seen. By contrast, the scratch closure in the maleimide-EGF/Gel group was not significantly different from that in the control group. After 24 h, the scratch closure rate in the EGF/Gel group was the highest and almost complete. There was no further increase in the EGF/Gel group after 24 h because the scratch closure was completed. The closure rate of the maleimide-EGF/Gel group increased after 24 h and the value increased significantly compared to that of the control group but was still lower than that of the EGF/Gel group. After 48 h, the scratch closure rate of the maleimide-EGF/Gel increased significantly, and the closure was almost complete. The scratch closure was completed in 24–48 h in the scratch assay; thus, it was difficult to observe any long-term effects beyond this time point. In a practical environment, such as tissue wound healing, it is expected that the effect of maleimide-EGF will continue for a longer period.

Overall, a sustained-release system was developed by introducing a maleimide group to EGF and chemically bonding it with the furfuryl-gelatin hydrogel. The maleimide group of EGF was combined with the furfuryl group of gelatins using the Diels–Alder reaction. The EGF, which is physically entrapped in the hydrogel matrix, showed an initial burst release with most of the EGF being released during the first two days. However, maleimide-EGF, which is chemically bound to the matrix, exhibited a release behavior similar to the gelatin hydrogel degradation. The maleimide-EGF was released slowly over seven days. The physical and mechanical properties of the furfuryl-gelatin hydrogels can be controlled by varying the concentration of the precursor solution. For biomedical applications, the degradation rate and mechanical properties can be determined based on the crosslinking density. Moreover, the introduction and release of biomolecules can enhance the versatility of injectable in situ gelatin hydrogels in tissue engineering.

## 3. Conclusions

In this study, the promising application of furfuryl-gelatin hydrogel was investigated by controlling its mechanical properties and growth factor delivery. Riboflavin phosphate was used as the photoinitiator and visible light as the light source. The range of adjustable storage modulus was investigated by varying the concentration of furfuryl-gelatin hydrogel, allowing us to investigate its effects on cells because of their mechanical properties. In addition, we demonstrated that introducing the maleimide group to a therapeutic factor with a small size allowed sustained release in accordance with hydrogel matrix degradation without initial burst release. In the present study, gelatin hydrogel and EGF were used as the matrix and the therapeutic factor, respectively. The NHS ester coupling reaction for introducing a furfuryl group can be used for various biomolecules containing an amine group. Owing to numerable possible variations, these modified hydrogels can be widely used in the biomedical field.

## 4. Materials and Methods

### 4.1. Materials

All the chemicals and solvents were used as provided by the manufacturer. Gelatin (gelatin powder from bovine skin), furfuryl glycidyl ether (96%), dimethyl sulfoxide (DMSO), deuterium oxide (D_2_O, 99.9% atom% D), riboflavin 5′-phosphate sodium salt hydrate (riboflavin phosphate), and the Pur-A-Lyzer™ Maxi Dialysis Kit were purchased from Sigma-Aldrich (St. Louis, MO, USA). Fetal bovine serum (FBS), phosphate-buffered saline (PBS, pH 7.4), Dulbecco’s phosphate-buffered saline, 3-(4,5-dimethylthiazol-2-yl)-2,5-diphenyltetrazolium bromide (MTT), Coomassie Plus (Bradford) Assay Kit, and recombinant murine EGF were purchased from Thermo Fisher Scientific (Waltham, MA, USA). Ethyl ether, 1 N sodium hydroxide (NaOH), 1 N hydrochloric acid (HCl), and acetone were purchased from Duksan Pure Chemical Co. Ltd. (Seoul, Korea). Dulbecco’s modified Eagle’s medium (DMEM), penicillin–streptomycin, and trypsin-EDTA solution were purchased from WelGene, Inc. (Daegu, Korea). NIH/3T3 cells were procured from the Korean Cell Line Bank (Seoul, Korea). The mouse ELISA kit was purchased from Komabiotech (Seoul, Korea). Maleimide-PEG_2_-NHS ester was purchased from Tokyo Chemical Industry Co. Ltd. (Tokyo, Japan). Cell scratch inserts were purchased from IBIDI GmbH (Munich, Germany).

### 4.2. Synthesis and Characterization of Furfuryl-Gelatin

Furfuryl-modified gelatin was synthesized through a coupling reaction between the amine groups of gelatin and furfuryl glycidyl ether. First, gelatin (2 g) was dissolved in 80 mL of deionized (DI) water and the pH was adjusted to 11 using 1 N NaOH. Next, furfuryl glycidyl ether (250 μL) was mixed with DMSO (20 mL). Then, these two solutions were mixed and incubated at 60 °C for 30 h with gentle stirring. The pH of the furfuryl-gelatin solution was adjusted to 7 and dialyzed for 48 h using a dialysis membrane (MWCO 1000 Da, Spectrum Laboratories, Inc., Rancho Dominguez, CA, USA). The dialyzed furfuryl-gelatin solution was lyophilized and then washed four times with acetone and once with ether. The solution was then dried at 37 °C for 6 h and stored for further use.

The synthesized furfuryl-gelatin was analyzed using ^1^H-NMR (500 MHz) using JNM-ECZ500R/S1 (JEOL, Tokyo, Japan). Gelatin and furfuryl-gelatin were dissolved in D_2_O (50 mg/mL) for the measurements.

Fourier-transform infrared (FT-IR) spectra were obtained at room temperature using an iS50 spectrometer (Thermo Fisher Scientific, Waltham, MA, USA). The transmittance of the lyophilized samples was measured using KBr pellets within the range of 4000–500 cm^–1^.

### 4.3. Fabrication and Characterization of the Crosslinked Furfuryl-Gelatin Hydrogel

Furfuryl-gelatin hydrogels were prepared by dissolving lyophilized furfuryl-gelatin in PBS at 10, 20, and 30% (*w/v*). Riboflavin phosphate was then added to the furfuryl-gelatin solution at 0.01 % (*w/v*). Finally, crosslinking was induced by irradiating the hydrogel precursor solution with visible blue light (458 nm) for 10 min.

Scanning electron microscopy (SEM) analysis was performed using an S-4700 (Hitachi, Tokyo, Japan) at the Smart Materials Research Center for IoT, Gachon University, South Korea. The furfuryl-gelatin hydrogel samples were incubated in DI water for 24 h and freeze-dried, and then, the cross sections of the hydrogels were imaged.

The swelling ratio of the hydrogel was calculated using the weight ($W_{d}$) of 100 µL of the hydrogel after freeze-drying for 48 h and the weight $(W_{s}$) after swelling in PBS for 24 h. It is quantified using following equation:

(2) $$\mathrm{Swelling}\;\mathrm{ratio}:\;\frac{W_{s}-W_{d}}{W_{d}}\times 100\left(\%\right)$$

The dynamic rheological behavior of the hydrogels was measured with a rheometer (MCR92, Anton Paar, Graz, Austria) using a 25-mm diameter, parallel geometry, and transparent bottom plate. The time and frequency sweep measurements were performed at 37 °C. For measuring the dynamic modulus as a function of time, 10% furfuryl-gelatin hydrogel precursor solution was mounted on the rheometer plate. The storage (G’) and loss (G’’) moduli were measured at a fixed strain (1%) and frequency (1 Hz) by irradiating the blue light for 10 min through the transparent bottom plate. For measuring the dynamic modulus as a function of frequency, the 10%, 20%, and 30% furfuryl-gelatin hydrogels were first fabricated and incubated at 37 °C for 3 h to complete crosslinking. The hydrogels were then mounted on the sample stage of the rheometer, and G’ and G’’ were measured at a fixed strain (1%) in the linear viscoelastic region, which was previously assessed using strain sweep experiments.

The thermal decomposition behavior of the hydrogels was measured using a thermogravimetric analyzer (Perkin Elmer, Waltham, MA, USA). The hydrogel sample (10 mg) was placed in an open aluminum pan for the analysis, which was performed in the range of 20 to 500 °C using 10 °C/min temperature rise.

The degradation of the furfuryl-gelatin hydrogel was analyzed by measuring the amount of gelatin in the incubated solution via protein quantification. The furfuryl-gelatin hydrogel was incubated at 37 °C, and the PBS solution was replaced daily. Protein quantification was performed using the Bradford assay following the manufacturer’s protocol.

### 4.4. Modification and Characterization of EGF with the Maleimide Group

EGF was prepared as a 1 mg/mL solution in PBS. Then, 20.6 mg/mL of maleimide-PEG_2_-NHS ester was dissolved in DMSO. These two solutions were then mixed at a 9:1 volume ratio, and the reaction was allowed to proceed for 2 h at 4 °C. To remove the unconjugated maleimide-PEG_2_-NHS ester, the mixed solution was dialyzed using a dialysis tube (MWCO 6000 Da). Dialysis was performed at 4 °C using PBS which was constantly replaced at fixed intervals of 1, 2, 3, and 6 h. After dialysis, the solution was subdivided and frozen.

The resultant maleimide-PEG_2_-conjugated EGF (maleimide-EGF) was analyzed using matrix-assisted laser desorption and ionization time-of-flight (MALDI-TOF) mass spectrometer (Autoflex maX, Bruker, Billerica, MA, USA) at Gyeonggido Business & Science Accelerator, South Korea.

### 4.5. NIH/3T3 Fibroblast Culture and MTT Assay

NIH/3T3 fibroblasts were cultured in culture medium (DMEM supplemented with 10% *v/v* FBS and 1% *v/v* penicillin–streptomycin) and incubated at 37 °C in 5% CO_2_ and 95% air.

The bioactivity of maleimide-EGF was evaluated using the MTT assay. The cells (5 × 10^4^ cells) were seeded into each well of a 24-well plate. The culture medium was added to the cells of the control group. EGF and maleimide-EGF as 1 and 10 µg/mL, respectively, were added to the culture media as well as cells to determine the effect of the maleimide group. The MTT assay was performed after 24 h of incubation. Subsequently, the culture medium was removed, and 10% *v/v* MTT solution (6 mg/mL) was added. The cells were incubated for 1 h at 37 °C, and the formazan crystals formed from MTT by mitochondrial reductase were dissolved in DMSO. Finally, absorbance was measured at 540 nm using a microplate reader (Agilent Technologies, Santa Clara, CA, USA).

### 4.6. Fabrication of EGF-Containing Furfuryl-Gelatin Hydrogel and EGF Release

The EGF-containing hydrogel was prepared by mixing the EGF and furfuryl hydrogel precursor solutions. First, EGF/Gel and maleimide-EGF/Gel groups were prepared by adding 1 μL of EGF or maleimide-EGF solution (0.01 mg/mL) to 100 µL of the furfuryl-gelatin hydrogel precursor solution, respectively. Then, the solutions were incubated at 60 °C for 15 min to combine the maleimide and furfuryl group. Finally, the prepared solutions were irradiated by blue light.

The fabricated EGF/Gel and maleimide-EGF/Gel was incubated in 1 mL of PBS at 37 °C. The PBS solution was replaced, and the cells were collected daily. The EGF or maleimide-EGF in the collected PBS solution was quantified using an EGF ELISA. The EGF ELISA kit protocol was followed, and the absorbance of each well was measured at 450 nm using a microplate reader. For quantification of EGF and maleimide-EGF, standard curves for both were obtained, and subsequently, the amounts of EGF and maleimide-EGF were calculated.

### 4.7. Fibroblast Proliferation and Scratch Assays

The NIH/3T3 fibroblasts (5 × 10^4^ cells) were seeded in each well of 24-well plates and the fabricated hydrogel was added to the well plates. Cell proliferation was measured using the MTT assay on days 1, 3, 5, 7, and 10 using the protocol described earlier.

A scratch wound-healing assay was then performed to evaluate cell migration/proliferation. NIH/3T3 fibroblasts (3.5 × 10^3^ cells) were seeded on each side of the cell followed by scratching, inserting, and incubating overnight until an optically confluent monolayer was formed. The gap closure was observed using an optical microscope (EVOS M5000 Imaging System, Thermo Fisher Scientific, Waltham, MA, USA), and images were taken at 0, 12, 24, and 48 h for each group. The remaining scratch areas were quantified using the ImageJ wound-healing measurement tool. The scratch closure was then assessed by dividing the remaining open area by the initial scratch area.

### 4.8. Statistical Analyses

All data are expressed as the mean ± standard deviation, and each experiment was repeated thrice unless indicated otherwise. Statistical analyses were performed using one-way analysis of variance with the GraphPad Prism 9 software. *p* < 0.05 indicated significant differences.

## Data Availability

The data generated or analyzed during this study are available from the corresponding authors on reasonable request.
